# Supplementary material for: Are carbon emissions trading and green financial instruments synergistic? -Comprehensive quantitative research based on content analysis
Source: PLoS One. 2024 Mar 7;19(3):e0298601. doi: 10.1371/journal.pone.0298601 (PMC10919601; doi:10.1371/journal.pone.0298601)
Supplement: S1 Data — (ZIP) [file pone.0298601.s001.zip › Data/地级市Z_F工作报告.zip!/║╙─╧/─╧╤⌠╩╨/└┤╫╘╣1⁄2╓┌║┼í╛┬φ┐╦╩2╛▌═°í┐_2009.docx]

工作回顾

综合实力大幅提升经济发展成效显著。去年全市生产总值完成1636.4亿元是2003年的2.3倍年均增长13.6%。三次产业结构由"二一三"升级为"二三一"。地方财政一般预算收入51.3亿元是2003年的2.4倍。全社会固定资产投资895.8亿元是2003年的4.3倍一大批重点项目相继建成。工业经济提速增效规模以上工业增加值、实现利税分别是2003年的3.6倍和4.8倍12个优势产业完成增加值占全市规模以上工业的80%以上一批特色产业园区发展壮大年销售收入超10亿元企业达17家新创中国驰名商标2个、中国名牌5个。自主创新能力进一步增强我市被确定为新能源产业国家高技术产业基地、河南省光电产业基地、河南省生物产业高技术产业基地。文化旅游产业快速发展五年累计接待游客3367万人次、实现旅游综合收入179.4亿元。南阳伏牛山被评为世界地质公园。环境保护和节能减排工作力度加大矿产企业整合和节约集约用地取得明显成效我市被确定为全国首批可持续发展试点城市。

"三农"工作全面加强农村面貌发生较大变化。认真落实各项支农强农政策全部取消农业税累计发放各类涉农补贴资金38.2亿元。粮食总产稳定增长达到114亿斤。油料总产突破百万吨大关。烟叶生产质量效益均创新高被确定为全国优质烟科技示范基地。畜牧业产值占农业总产值的比重达到34%。9个土特产品被认定为中国地理标志产品。全市提前一年实现平原绿化高级达标森林覆盖率由28.8%提高到36%。农业产业化步伐加快。农村生产生活条件明显改善连年夺得省"红旗渠精神杯"新建农村沼气用户33万户解决了81.6万农村居民饮水安全问题减少贫困人口57.3万人实现所有行政村通电、通油路(水泥路)、通电话、通宽带、通广播电视。南水北调中线工程南阳段开工建设库区移民试点工作全面启动。新农村建设稳步推进培育了一批亮点和典型。

基础设施不断完善城镇化步伐加快。中心城市建成区面积由五年前的55平方公里扩展到90平方公里。完成了一大批路、桥、坝新建改建工程和背街小巷整治工程。城中村改造扎实推进。城市环卫和城管体制改革顺利完成。荣获"中国优秀旅游城市"、"国家园林城市"称号。县城和小城镇建设迈出新步伐全市城镇化率由25.5%提高到34.9%。交通建设加速推进被交通运输部确定为国家级公路运输枢纽城市高速公路从无到有总里程达553公里居全省第一位新建改建干线公路1895公里、县乡公路3114公里、村道14181公里宁西铁路客运通车南阳机场直航线路增至6条。

改革开放深入推进发展活力明显增强。国有企业改革成效显著38家市属工业企业改制大头落地。义务教育管理、粮食流通、水管等体制改革相继完成行政审批、集体林权等制度改革取得明显成效交通、建设、供销、公用事业等领域改革迈出新步伐。企业上市实现零的突破新纺、利达光电成功上市。中小企业信用担保体系基本建立。非公有制经济快速发展占生产总值的比重达到56.2%五年提高12.9个百分点。大力引进战略投资者企业战略重组迈出重大步伐。成功举办了四届玉雕节、五届张仲景医药文化节、第三届豫商大会和首届宝玉石博览会积极组织参加中原文化港澳行等一系列大型经贸招商活动累计引进市外资金570亿元。五年进出口总额完成25亿美元年均增长31.6%。批准外商投资企业160家实际利用外资3.5亿美元、年均增长27.2%。

致力解决民生问题人民生活持续改善。城镇居民人均可支配收入、农民人均纯收入分别达到12395元和4570元均比2003年增加1倍以上2008年末城乡居民储蓄存款余额达687.4亿元是2003年的2倍。五年新增城镇就业57万人、农村劳动力转移就业75万人零就业家庭实现动态归零城镇登记失业率控制在3.8%以内荣获"全国就业促进活动先进单位"称号。城镇职工基本养老、医疗、失业等社会保险覆盖面不断扩大。建立完善了农村低保制度城市低保标准不断提高。全市城镇医保参保居民达60.3万人新农合参合率达到95.5%覆盖城乡的医疗保障体系框架基本形成。建设经济适用住房71.9万平方米建立了廉租住房保障制度。大力实施食品放心工程食品安全水平不断提高。

经济社会协调发展各项社会事业全面进步。科技对经济增长的贡献率达到47%荣获"全国科技进步先进市"称号。教育投入不断增加"两基"工作通过国检认真落实"两免一补"政策全面实行城乡免费义务教育。农村办学条件明显改善圆满完成农村中小学校D级危房改造、远程教育工程建设等任务。大力推进中心城市义务教育均衡发展新建、改扩建中小学校26所新增学位2.2万个缓解了"大班额"和上学难问题。职业教育加快发展助学制度进一步完善。高等教育规模不断扩大南阳理工学院升格为本科院校南阳医专、河南工业职业技术学院挂牌成立南阳师院和南阳医专先后通过教育部评估验收。公共卫生服务体系进一步健全市县疾病防控中心和医疗救治项目建成投运县乡村三级预防医疗网络建设得到加强被授予"全国农村中医工作先进市"。文化事业繁荣发展内乡宛梆等8个项目被列入国家非物质文化遗产名录荣获"中国楹联文化城市"称号。有线电视数字化整体平移工作进入全国先进行列。成功申办2012年全国第七届农民运动会。人口和计划生育工作新机制不断完善低生育水平持续稳定被确定为全国计划生育综合改革试点市。人事、统计、规划、物价、新闻出版、工商、税务、金融、口岸、供销、农机、盐业、史志、气象、地震、人防、消防、对台、侨务、外事、民族宗教、残疾人以及民兵预备役、"双拥"等工作都取得了新的成绩。

精神文明和民主法制建设明显加强和谐南阳建设扎实推进。深入开展社会主义荣辱观教育和精神文明创建活动广大干部群众思想文化素质和精神风貌发生新的变化。"五五"普法全面启动法律服务体系日益健全。认真贯彻落实《全面推进依法行政实施纲要》和《行政许可法》政府依法行政能力不断增强。加强政府廉政建设和效能建设强化行政监察和审计监督政风行风明显好转。积极推行政府信息公开。应急管理体制建立运行。自觉接受人大法律监督和政协民主监督五年共办理人大代表建议和政协委员提案3690件办结率100%、满意率97.6%。全面推进基层民主政治建设顺利完成第五、第六届村委会换届选举城镇社区建设加速推进。重视加强人民群众来信来访工作深入开展"平安南阳"创建活动狠抓安全生产社会大局保持和谐稳定。全力支援四川抗震救灾圆满完成抢险救援和过渡房建设任务对口援建工作走在全省前列。

各位代表过去五年是我市综合实力提升快、社会建设成效好的五年是人民群众受益多、城乡面貌变化大的五年是奠定坚实基础、积蓄发展后劲的五年是我市经济社会发展最好最快的时期之一。五年来取得的巨大成就是省委、省政府和市委正确领导的结果是市人大、市政协大力支持的结果是全市人民共同奋斗的结果。在此我代表市人民政府向全市广大干部群众向市人大代表、政协委员、各民主党派、工商联、各人民团体、离退休老同志、无党派人士向驻宛企事业单位、驻宛部队、武警官兵向所有关心支持南阳发展的各界朋友表示崇高的敬意和衷心的感谢!

各位代表总结回顾过去五年的探索与实践以下五条经验弥足珍贵需要我们认真把握和长期坚持一是必须把解放思想作为应对新挑战、开创新局面的一大法宝与时俱进开拓创新奋发有为二是必须把加快发展作为第一要务以科学发展观统领全局强化项目支撑加快工业化、城镇化进程带动经济社会全面振兴三是必须把改革开放作为强大动力重视营造发展环境以开放的思维、改革的办法破解难题、促进发展以良好的环境增强吸引力、竞争力四是必须把改善民生作为政府工作的出发点和落脚点坚持以人为本让改革发展成果更多地惠及于民五是必须把务实重干作为基本要求牢记使命求真务实多做打基础、增后劲、管长远的事以事业凝聚人心以发展鼓舞士气。

在肯定成绩的同时我们也清醒地认识到全市经济社会发展还面临不少困难和问题。主要表现在五个方面一是经济实力大而不强。我市主要经济指标人均水平在全省长期处于靠后位次财政收入占生产总值的比重仅为全省平均水平的二分之一。二是经济结构不合理经济发展方式粗放。农业基础还比较脆弱工业经济不大不强第三产业比重偏低节能减排任务艰巨土地、资源、环境等约束加剧城镇化水平低中心城市辐射带动能力弱县域经济发展整体水平有待提升。三是体制机制性矛盾仍很突出。政府机构改革有待深化事业单位改革进展缓慢财政供养人员过多、包袱沉重国有商贸流通企业、集体企业改革滞后、任务艰巨。四是和谐社会建设任重道远。社会保障和就业压力加大教育、医疗、社会治安等方面还有很多亟待解决的问题。五是政府自身建设有待加强。政府职能和作风转变不到位有的部门和工作人员责任意识、服务意识不强形式主义、官僚主义问题比较突出发展环境不优。对此我们一定要高度重视认真加以解决。

折叠编辑本段目标任务

站在继往开来的历史节点审时度势南阳到了奋力爬坡、跨越发展的重要关头。

我们面临的挑战前所未有。国际金融危机仍在扩散和蔓延冲击影响程度和持续时间难以预测。长期积累的体制机制性、结构性深层次矛盾和困难在新形势下逐步显现对改革发展稳定形成了沉重压力。区域竞争日趋激烈前边标兵越跑越远后边追兵日益迫近我们面临不进则退、慢进掉队的严峻挑战。面对崇高的历史使命面对全市1100万人民奋发图强的殷切期望我们必须增强忧患意识、危机意识和责任意识迎难而上以破釜沉舟的决心和气魄采取超常规举措实现跨越式发展。

我们面临的机遇和条件也前所未有。一是国际金融危机增强了国内外产业梯度转移的趋势有利于南阳在对内对外开放中寻求新的突破和更大发展危机形成的倒逼机制压力有利于我们加快结构调整和产业升级。二是国家实施积极的财政政策和适度宽松的货币政策加大对中西部和"三农"及社会建设的支持力度省委、省政府把南阳确定为区域性中心城市和省次中心城市有利于我们争取更多、更大的支持解决自身难以解决的问题。三是南阳自然资源丰富文化积淀深厚区位优势日益凸显正处于工业化、城镇化加速发展时期基础设施建设、产业振兴、社会发展等方面有巨大空间。四是南水北调中线工程南阳段、南阳核电站、南阳粮食主产区建设和全国第七届农运会筹办等重大工程陆续上马多年来谋长远、打基础、增后劲积蓄的巨大能量与全市上下人心思进的精神动力融合在一起必将为我们应对挑战、实现跨越式发展提供强大的物质和信心支撑。

今后五年政府工作的总体要求是以科学发展观统领经济社会发展全局实施"三大战略"奋力实现"四大突破"加快构建现代产业体系、现代城镇体系和自主创新体系努力推动经济大市向经济强市跨越、文化旅游生态资源大市向文化旅游强市和生态宜居名市跨越为中原崛起做出重要贡献。

今后五年经济社会发展的主要奋斗目标是到2013年全市生产总值、地方财政一般预算收入、全社会固定资产投资在优化结构、提高效益、降低消耗、保护环境的基础上力争比2008年翻一番实现五年倍增。主要经济指标年均增速达到、力争超过全省平均水平。城镇化率达到45%年均增长2个百分点。这些奋斗目标是按照上述总体要求把南阳置身于周边、全省乃至全国的发展大局中衡量在综合分析形势、认真研究市情的基础上确定的。这些目标是时代的要求、人民的期盼非常必要我们一定要努力实现!这些目标也是切实可行的我们坚信经过全市人民的共同奋斗一定能够实现!

根据上述总体要求和奋斗目标今后五年要突出抓好以下六个方面的工作

(一)建设新型工业强市构建现代产业体系。实施工业强市战略走新型工业化道路加快构建以高新技术产业为先导、以先进制造业和现代服务业为主体、以大型骨干企业为支撑、以产业集聚区为载体的现代产业体系努力在培育壮大战略支撑产业上取得突破性进展。力争到2013年生物、光电、光伏、超硬材料、石油化工、碱硝化工、先进制造、纺织、冶金、建材、医药、电力等12个优势产业销售收入均突破100亿元重点打造的新能源基地、新材料基地和食品产业基地分别达到600亿元、400亿元、500亿元以上产业集聚区工业增加值突破100亿元的达到10个2个达到300亿元以上销售收入50亿元-100亿元的企业达到10家2家达到200亿元以上。把服务业放在更加突出的战略位置加强规划引导加大政策扶持突出发展现代物流、通信、金融、商务等生产性服务业培育提升房地产、餐饮、社区服务等消费性服务业培育壮大一批现代物流园区和企业集团力争服务业占生产总值的比重每年提高一个百分点以上。

(二)加快建设区域性中心城市统筹城乡协调发展。实施中心城市带动战略构建中心城市、中小城市、小城镇协调发展的现代城镇体系。做大做强中心城市。围绕建设区域性中心城市目标加强中心城市发展战略研究按照"白河为轴、两岸并举、区块突破、组团发展"的思路坚持城市建设与产业发展、文化建设、生态建设并重强化枢纽地位扩大城市规模增强城市功能壮大城市经济改善城市环境全面提升城市综合承载力和区域发展服务能力努力把南阳建设成为全省全国知名的新型工业强市、文化旅游强市和生态宜居城市成为带动全市、辐射周边、助推中原崛起的区域性中心城市。力争到2013年中心城区建成区面积达到120平方公里人口120万人中心城市经济总量占全市的比重大幅度提升。向心发展加快构建现代城镇体系。制订实施南阳现代城镇体系发展规划和配套政策积极构筑以南阳中心城市为核心以鸭河口、官庄工矿区为两翼以镇平、邓州、新野、内乡、方城、唐河、社旗、南召等城镇为环绕核心的城镇圈以宁西铁路、焦枝铁路和许南襄、沪陕高速公路两个"十字"形重要通道为市域城镇与产业发展轴以邓州市和桐柏、西峡、淅川县城为四极以小城镇为依托拓展完善公路、铁路、航空现代立体交通支撑体系形成"一体两翼、一圈两轴四极"向心发展、合理分工、功能互补、协调推进的城乡统筹发展新格局。发展壮大县域经济。研究出台促进县域经济发展的政策措施鼓励基础较好的县市率先发展、加速增强综合实力积极扶持相对落后地区发展力争1-2个县市进入全省县域经济综合实力十强。

(三)巩固农业基础地位促进农村经济繁荣发展。高度重视"三农"工作加大政策扶持力度加快农业基础设施和新农村建设促进农业增效、农民增收、农村繁荣。以建设南阳粮食主产区为契机稳步提高粮食生产力争到2013年粮食总产达到120亿斤。按照高产、优质、高效、生态、安全的要求切实抓好优质粮食、棉花、烟叶、油料、蔬菜、小辣椒、中药材、林果、花卉、食用菌十大主导产业基地建设和以南阳肉牛、生猪、奶牛为重点的优质畜产品生产加工基地建设推动南阳由传统农业大市向现代农业强市转变。增加、整合支农资金强化农业基础设施建设加大农综开发、扶贫开发力度提高农业机械化装备水平五年内使农业生产条件明显改善、综合生产能力明显提高完成大中小型水库除险加固、南水北调中线工程征地拆迁和移民安置任务解决农村饮水安全问题和现有全部贫困人口的脱贫问题。按照新农村建设"二十字"方针要求以试点村、示范村建设和特色产业培育、村容村貌整治为重点因地制宜整村推进力争到2013年全市新农村建设实现较大跨越农村面貌显著改善、农民生活更加宽裕。

(四)发挥资源优势加快建设文化旅游强市和生态宜居名市。坚持市场化、产业化、品牌化运作和规模化、集约化发展深入挖掘开发南阳楚汉文化、医药文化、衙署文化、曲艺文化、玉文化、盘古文化、赊店商业文化等特色文化保护开发文化名胜和文化遗产加快发展特色文化产业、新兴文化产业和社会文化产业构筑主业突出、结构合理、特色鲜明的文化产业体系大力推进文化事业单位改革和文化管理体制改革鼓励各类资本进入政策许可的文化领域促进文化资源大市向文化强市转变。围绕"游在伏牛、养在南阳"目标积极创建旅游强市和生态宜居名市。坚持在保护中开发、在开发中保护以伏牛山生态游为龙头创建一批山水精品培育一批文化名片重点打造宝天曼、老界岭、南水北调中线渠首、恐龙遗迹园、玉雕湾、山陕会馆等核心景区完善旅游线路强化宣传推介提升服务质量拉长产业链条促进旅游业快速发展。充分开发利用南阳丰富、地道的中药材资源坚持医教研、产加销六位一体弘扬张仲景中医药文化致力打造中医药都和养生胜地。实施碧水蓝天工程强力推进节能减排加大污染治理力度搞好造林绿化强化生态保护努力把南阳建成山水园林相间、生态环境优美、人与自然和谐发展的宜居健康家园。

(五)大力实施开放带动和创新推动战略增强发展动力活力。把开放带动作为跨越发展的主战略依托优势资源和骨干企业办好节会、园区载体积极承接产业转移着力引进国内外大企业、大财团等战略投资者重点谋划引进一批超亿元、超十亿元的大项目。调整优化出口产品结构扩大横向经济技术合作不断拓展双向开放的广度和深度。优化经济发展环境着力打造"零障碍、低成本、高效率"的投资环境。深入推进各项改革。积极实施、如期完成新一轮政府机构和乡镇机构改革。巩固扩大国有工业企业改革成果。加快商贸流通企业、集体企业改革步伐全力推进事业单位改革争取五年内完成改制任务。大力开展全民创业出台优惠政策激活各类创业主体支持中小企业做大做强。积极推进财税、金融等重要领域和关键环节的改革攻坚尽快形成有利于推动跨越发展的体制机制。深入实施科教兴宛和人才强市战略加快构建以企业为主体、以市场为导向、产学研相结合的自主创新体系。力争2013年全市高新技术产业增加值占规模以上工业增加值的比重达到30%自主创新能力达到全省先进水平。

(六)加强社会建设努力构建和谐南阳。扎实推进以改善民生为重点的社会建设使广大人民群众学有所教、劳有所得、病有所医、老有所养、住有所居共享改革发展成果。坚持教育优先发展战略优化资源配置实现义务教育均衡发展基本普及高中阶段教育大力发展职业教育提升高等教育发展水平。进一步完善卫生服务体系基本建立覆盖城乡居民的基本医疗卫生制度为群众提供安全、有效、方便的医疗卫生服务。积极开展全民健身活动提高竞技体育水平办好第七届全国农运会。加强就业服务体系建设改善就业环境促进社会就业。完善养老、医疗、失业、工伤、生育保险制度和城乡社会救助制度全面提高社会保障水平。加大经济适用住房和廉租住房建设力度致力解决城市低收入家庭住房困难问题。强化社会建设和管理营造社会长治久安、人民安居乐业的良好环境。

折叠编辑本段工作安排

2009年是新一届政府的开局之年也是经济增长下行压力加大、发展较为困难的一年。今年以来国内外形势变化对我市的影响日益加重主要经济指标增长速度全面下滑亏损企业亏损额大幅度增加经济形势异常严峻。我们必须保持清醒头脑积极应对宏观经济环境变化变压力为动力化挑战为机遇振奋精神扎实工作努力保持经济平稳较快发展为全面完成未来五年目标任务打下坚实基础。

今年全市经济社会发展的主要目标是生产总值增长11%地方财政一般预算收入增长9.5%全社会固定资产投资增长20%以上外贸出口增长10%实际利用外资增长10%城镇居民人均可支配收入实际增长7%农民人均纯收入实际增长6%城镇登记失业率控制在4%以内人口自然增长率控制在6.5%。以内单位生产总值能耗降低5.1%化学需氧量、二氧化硫排放总量分别控制在6.86万吨和6.68万吨以下。重点抓好以下六个方面的工作

(一)扩大投资和消费需求保持经济平稳较快增长

着力扩大投资需求。一是加快大型骨干项目建设。启动实施"四大工程"。南水北调中线工程扎实做好征地拆迁安置和协调工作加强与移民迁入地的对接完成1.06万库区移民试点安置任务确保南水北调中线工程南阳段顺利实施。南阳核电工程完成核电站址搬迁群众安置新村建设任务和600万方土石方平整工作实现站址"四通一平"保证核岛混凝土按期顺利施工。南阳粮食主产区工程着力抓好优质粮产业工程及农机装备工程加快中低产田改造推进大中型水库除险加固和鸭灌、引丹等大中型灌区续建配套及节水改造。全国农运会筹备工程搞好竞赛场馆规划设计开工建设一批功能性工程。在实施"四大工程"的同时在能源、交通、农业、民生、城建等方面规划实施305个重大投资项目确保中心城区污水管网等项目开工建设南阳机场航站楼等项目竣工投运大力支持平顶山-南阳白河双回路输变电工程、西气东输二线长输管线等重点项目建设做好天池抽水蓄能电站、鸭电三期、白河南热电厂、方城风电二期、宁西铁路复线、内邓高速、南邓一级公路、社旗至南阳一级公路等重大项目前期工作。二是积极争取上级投资。抓住国家扩大内需的政策机遇筛选论证一批重大项目切实做好争取工作。加强中央投资项目监管致力建设优质工程、惠民工程、廉洁工程。三是努力推动社会投资。深化投融资体制改革拓宽投融资渠道和领域。发挥财政杠杆作用加大贴息和补助力度引导和扩大社会投资。

努力扩大消费需求。把调整收入分配、发展服务业与扩大消费结合起来大力开拓消费市场。增强居民消费能力。积极推进事业单位工资制度改革落实企业最低工资制度和企业退休人员待遇政策健全农民工工资支付保障机制。提高优抚对象、建国前老党员等抚恤和生活补助标准加大惠民政策扶持力度逐步提高对低收入家庭的补贴和补助努力增加城乡居民收入。积极培育消费热点。扩大农村消费、住房消费、文化旅游消费和服务消费。继续实施"万村千乡市场工程"、"双百市场工程"搞好农村便民超市建设和农产品批发市场升级改造发展农资连锁配送构建农村现代流通网络。增加家电下乡补贴品种引导农民扩大消费需求。积极鼓励住房消费引导房地产企业多开发建设中低价位的普通商品房鼓励大型房地产企业进行集中连片开发促进房地产市场健康稳定发展建设普通商品住房270万平方米。大力发展文化旅游产业推动文化旅游消费全年实现游客接待量力争达到1200万人次、旅游综合收入60亿元。加快发展住宿餐饮、社区商业、物业、家政等生活性服务业引导居民扩大消费。优化消费环境。研究出台鼓励消费的政策发展和完善消费信贷。加强市场监管整顿和规范市场秩序坚决打击制售假冒伪劣商品和价格欺诈等违法行为维护消费者合法权益。

(二)大力实施工业强市战略

加快工业重点项目建设。全年计划实施重大项目121个确保防爆集团大型同步电动机等52个项目竣工投产二胶厂PCB胶片生产线等57个项目开工建设。对50个工业"发动机计划"项目市财政安排1亿元资金予以扶持并在用地上优先保障促其尽快建成投产达产。继续开展"工业强市杯"竞赛活动鼓励支持各县市区重点抓好投资3000万元以上的工业项目推动工业经济快速发展壮大。

集中培育大型企业集团。以12个规划销售收入超百亿元的骨干企业或企业群体为依托通过技术创新、战略重组、政策扶持等措施集中培育一批主业突出、拥有自主知识产权和核心竞争力的大型企业集团。继续在全市工业企业中开展"十大纳税企业"、"十大高成长性企业"评选活动激励企业做大做强。重视加强企业经营管理人才培养努力造就一支境界高、素质好、富有开拓创新精神的企业家团队。

增强自主创新能力。加大政策扶持力度推动光电、光伏、生物能源、生物化工、超硬材料和先进制造业等高技术产业关键技术研发抓好光学引擎、多晶硅、超硬材料、纤维乙醇等高新技术产业化项目加强对外合作尽快扩大产能加快高新技术产业化步伐。鼓励扶持骨干企业与国内外行业重点企业、高等院校、科研院所合作支持重点实验室、企业技术中心、工程技术中心、博士后科研工作站建设开展科研攻关转化推广科技成果。集成科技资源力争激光电视、电子纸等国家"863计划"项目和核电级防爆电机、紫激光计算机直接制版(CTP)版材及制版机等重大科技专项实现新的突破。

大力发展产业集聚区。加快全市13个首批省定产业集聚区规划建设明确目标完善功能突出特色搞好各类生产要素的集约配置提高产业集中度和承载力把产业集聚区打造成为招商引资的主阵地、经济发展的重要增长极和现代化城市新组团。尽快做大做强高新区、白河生态园区和龙升工业园区搞好园区规划创新市区管理体制完善配套设施提升服务水平加大项目引进建设力度提高投资强度和产出效益努力实现更大规模、更高水平发展。

强力推进节能减排。全面落实节能减排目标责任制和"一票否决"制确保完成年度目标任务。突出抓好96家重点耗能企业的节能降耗推进余热余压利用等十大重点节能工程建设。加强城乡污染治理设施和减排工程运行监督确保已建成污水处理厂达标运营燃煤电厂脱硫设施投运率达到95%以上。搞好淅川钒土开采冶炼、丹江口库区等重点区域综合整治加强重点流域水污染防治和冶金、造纸、制药等行业废水深度治理。

(三)全面加强"三农"工作

稳定发展粮食生产调整优化农业结构。按照稳定面积、提高单产、增加总量、改善品质的原则大力发展粮食生产全年粮食保持在1600万亩以上总产达到100亿斤以上。在发展优质粮食、棉花、油料、蔬菜、烟叶等作物的同时加快发展中药材、食用菌、小辣椒、林果、花卉、茶叶、蚕茧等特色产业建立一批高标准、高质量的生产加工基地。大力发展规模养殖全年新发展300头以上肉牛育肥场40个、年出栏万头以上规模养猪场20个、200头以上奶牛养殖场15个。以开展"常青杯"竞赛活动为载体扎实搞好林业生态建设完成造林99.94万亩。加强农产品质量检测体系、动植物疫病防控体系、农业标准化体系、农业科技服务体系、农机服务体系、气象服务体系建设为发展现代农业提供重要保障。

大力推进农业产业化经营。把投资超5000万元以上的农业产业化龙头项目列入工业"发动机计划"确保科尔沁牛业年加工屠宰10万头肉牛项目一期工程、龙大牧原生猪分割生产线项目6月底前建成投产三色鸽乳业年产10万吨乳制品生产加工扩建项目12月份建成投产。加快农产品批发市场建设完善农村现代流通网络确保市级新合作配送中心和10个县级新合作配送中心规范运营。大力发展农民专业合作组织每个县市区新发展农民专业合作社20个以上。

扎实推进新农村建设和农业基础设施建设。继续开展"向荣杯"竞赛活动完成200个试点村、50个示范村建设任务、1782个村村容村貌整治任务。大力推进农村沼气建设新发展沼气用户10万户大中型沼气工程100座加强沼气社会化服务体系建设。积极开展"通达杯"竞赛活动搞好县乡公路建设和维护改建县乡公路1130公里、通村公路1200公里改造大中危桥6090延米。完成130个贫困村整村推进任务解决和巩固温饱人口11.17万人搬迁深山区贫困人口3000人以上。大力开展"兴水杯"竞赛活动新增有效灌溉面积13.5万亩、节水灌溉面积10.3万亩、旱涝保收田12万亩治理水土流失面积480平方公里解决25万农村人口饮水安全问题。加大农综开发力度完成20.8万亩中低产田改造任务。

多渠道增加农民收入。在挖掘农业内部增收潜力的同时鼓励农民发展多种经营创办"短平快"增收项目加快发展农村二三产业促进农民持续增收。坚持就地转移、异地输出与自主创业相结合多渠道开辟农民就业岗位。认真落实粮食直补等支农强农政策。稳定农业生产资料价格进一步减轻农民负担。

(四)加快城镇化进程

加大中心城市建设管理力度。一是强化规划的龙头作用。抓住全国第二次土地利用总体规划和省新一轮城市总体规划调整修编的机遇修订完善城市总体规划重点搞好控制性详规和各类专项规划做好产业集聚区规划实现控制性规划全覆盖。二是加强城市基础设施建设。加快独山大道南延、仲景南路续建工程进度新开工光武东路、光武西路、健康路、明山路改扩建工程对建设路、中州路、工业路部分路段进行大修改造。开工建设市博物馆、体育场、游泳馆、综合训练馆和市人防指挥所。加快仲景大桥建设进度开工建设光武大桥、雪枫大桥。抓好市污水处理厂二期、垃圾处理厂二期工程和滨河路污水管网建设。投资1000万元对城区54条背街小巷治理改造。加快规划建设东北分区和大学园区。优先发展城市公交增加公交线路和运营车辆适度扩大出租车运力方便群众出行。加强城市绿化建设启动汉宛城遗址、百里奚公园等一批公园、游园建设对城市主要道路、白河湿地公园绿化进行升级改造大力实施"一环一带四片六线"绿化工程重点抓好环城高速景观林带、兰营水库生态防护林工程和六大出入市口通道绿化美化工程。三是加快城中村改造和内河整治工程。对已启动实施的城中村改造项目加快改造进度搞好监督管理确保健康有序推进围绕白河两岸和内河两侧启动一批新的城中村和旧城改造项目。积极创造条件开工建设梅溪河整治工程搞好温凉河、三里河等内河整治前期设计。四是加强城市管理。深入开展"四城联创"和"靓丽杯"竞赛活动巩固扩大城管、环卫体制改革成果理顺城市管理体制建立城市管理长效机制推进城市标准化、精细化管理。加强社区建设规范物业管理夯实城市管理基础。

加快县城和小城镇建设。加大县城基础设施和公用设施建设力度逐步完善交通、通讯、供排水系统和生态保护系统不断完善城市综合功能。鼓励各县市开展"四城联创"活动进一步提升城市品位。大力发展特色小城镇和沿线、沿边城镇带开展示范城镇创建工作加快中心城镇发展步伐。

(五)深入推进改革开放

深化各项改革。加快国有工业企业改革完成28家国有工业企业改制遗留任务完善企业法人治理结构建立现代企业制度帮助企业尽快恢复生产。积极推进商贸流通企业和集体企业改革做好商场、五交化等5家企业改制工作加快黄牛清真、金汉丰等4家企业依法破产步伐。把推进企业上市作为产业结构优化升级、做大做强企业的战略举措强化对8家上市后备企业的培育和督导全力支持西泵公司等企业上市。积极稳妥地推进政府机构改革争取上半年完成市级政府机构改革下半年完成县级政府机构改革。加快推进事业单位改革步伐。搞好财税金融体制改革全面实施增值税转型、燃油税征收等税费改革。深化投融资体制改革进一步增强政府的融资和调控能力。推进农村综合改革探索建立农村土地承包经营权流转机制发展多种形式的土地适度规模经营完成集体林权制度改革任务。

扩大对外开放。研究出台招商引资激励政策进一步加大招商引资力度依托我市资源和产业优势策划包装一批招商项目重点面向国内外知名企业搞好推介。创新招商引资方式加快建立以企业和专业招商队伍为主体的招商机制推行招商引资目标管理扩大招商引资成果。抓好南阳A型保税物流中心建设力争年底前完成一期工程建设任务。加快企业战略重组步伐深化中光学集团与美国新泰辉煌公司、日本智能泰克株式会社金光数显公司与北京首钢股份公司利达光电公司与四川长虹电器公司天冠集团与中石油、内蒙古博源公司市政府与北方兵总、南方兵总、国家电网公司、中核集团、中电投、美国IDG公司、日本三菱公司的战略合作加快河南赊店酒业公司战略重组步伐支持淅川与湖北汉江集团合作组建新的电冶集团加快永煤集团钒系列产品项目进度支持洛钼集团收购整合镇平钼矿资源。精心组织各类经贸活动办好第二届宝玉石博览会和张仲景医药文化节组织好厦洽会、中博会、广交会等活动力争取得丰硕成果。坚持以质取胜和市场多元化战略千方百计扩大出口。

(六)扎实推进和谐社会建设

加大投入力度集中力量办好今年十大实事着力解决涉及群众切身利益的问题致力构建和谐社会。

千方百计扩大就业。实施更加积极的就业政策多渠道增加就业岗位。完善落实促进全民创业政策措施大力支持自主创业、自谋职业鼓励大中专毕业生、残疾人、退役军人和返乡农民工领办创办民营企业推动以创业促就业。积极推行农村劳动力技能培训、"阳光工程"和"雨露计划"提高农民工的劳动技能建立就业动态监控制度强化公共服务新增农村劳动力转移就业10万人。采取法律、行政等手段切实维护农民工合法权益。完善就业援助制度帮助城镇就业困难人员、零就业家庭和残疾人就业。全年新增就业8万人下岗失业人员再就业2.6万人其中就业困难对象再就业1万人零就业家庭保持动态归零。

进一步完善社会保障体系。推进社会保险扩面征缴清欠工作清欠率达到30%以上。重点做好非公有制企业从业人员、农民工、被征地农民和自由从业人员养老保险工作。完善失业保险制度确保失业保险金按时足额支付。将关闭破产企业退休人员和困难企业职工纳入城镇职工医保。做好农民工医疗和工伤保险工作参保人员分别达到2.3万人和7.5万人。巩固扩大城镇职工、居民基本医疗保险、生育保险和新农合医疗保障覆盖面积极探索推行新型农村社会养老保险制度。完善城乡社会救助体系和优抚保障制度提高城乡低保补助和农村五保集中供养标准。全面实施保障性安居工程全年建设廉租房18.1万平方米为11900户困难群众提供廉租住房保障建设经济适用住房52万平方米其中专项安排用于教师经济适用住房10万平方米。

大力发展社会事业。围绕实现教育公平整合教育资源优化教育布局加大教育投入促进各类教育均衡发展。全面落实义务教育各项优惠政策健全义务教育经费保障机制提高农村中小学公用经费标准改善农村办学条件。中心城区续建、改扩建、资源整合中小学校16所。重视学前教育规范农村幼儿教育努力提高城乡幼儿教育水平。加强职业教育实施示范性职业学校、薄弱职业学校、实训基地建设三大工程发展农村职业教育并逐步免费。整合玉雕职业教育资源加快建设镇平玉雕职业技术学院。加快发展高等教育启动南阳农校、南阳幼师、河南省经济管理学校升专工作。加强公共卫生和医疗服务体系建设完善农村三级医疗卫生服务网络搞好城市社区卫生服务体系建设加强疾病预防控制和卫生监督提高公共卫生服务能力和突发公共卫生事件应急能力。扶持中医药事业发展充分发挥中医药在防病治病中的重要作用。积极开展"曙光行动"为贫困白内障患者免费实施复明手术1万例。大力发展文化事业加强文化基础设施和文物保护工作实施文化惠民工程。广泛开展全民健身活动扎实开展第七届全国农运会筹备工作办好全市第三届运动会和首届农民运动会促进群众体育和竞技体育协调发展。加强人口和计划生育工作稳定现行的生育政策和低生育水平。

扎实做好安全生产和信访稳定工作。强化安全生产监督管理坚决遏制重特大安全事故发生。加强食品药品卫生安全监督管理确保人民群众饮食用药安全。加强和改进信访工作完善社会矛盾纠纷排查调处和防范化解机制健全信访工作责任制认真解决群众合理诉求有效控制群体性越级上访事件发生。深入推进"平安南阳"创建活动建立社会治安防控网络强化社会治安综合治理依法严厉打击各类违法犯罪活动始终保持对命案、黑恶犯罪、"两抢一盗"等犯罪活动的严打高压态势着力改善城乡社会治安状况提高人民群众安全感和满意度保持社会稳定和谐。

大力开展精神文明创建活动加强社会公德、职业道德、家庭美德和未成年人思想道德建设。重视国防教育支持驻宛部队、民兵预备役和人防建设深入开展"双拥"活动巩固发展军政军民团结创建国家双拥模范城市。充分发挥工会、共青团、妇联等人民团体的桥梁纽带作用。全面贯彻党的民族宗教政策广泛开展民族团结进步活动。扎实做好统计、审计、物价、工商、财税、金融、外事、侨务、对台事务、地方志、档案、气象、防震减灾等工作。继续抓好对口支援江油工作确保按期完成援建任务。

切实加强政府自身建设以学习实践科学发展观为动力深入推进"两转两提"努力建设人民满意的政府。

(一)建设责任政府。今后五年是南阳大发展的关键时期跨越发展的重任已经历史性地落在了我们肩上。各级政府领导干部要以强烈的危机意识、忧患意识以强烈的事业心、使命感始终抱着对南阳人民高度负责、对南阳发展高度负责、对南阳这一发展时期高度负责的态度始终保持昂扬向上的朝气、负重奋进的勇气、干就干好的决心切实担当起为官一任、造福一方的重任恪尽职守奋发有为力争使南阳经济社会发展有一个大的跨越。健全政府工作责任体系推行部门及岗位责任制明确职责和工作规程逐级分解目标确保责任到岗到人、任务落实到位。

(二)建设创业政府。实现今后五年奋斗目标必须扑下身子付出艰苦卓绝的努力。新一届政府要继承和发扬南阳先贤诸葛亮"鞠躬尽瘁、死而后已"的精神毫不懈怠、始终不渝地干事创业、艰苦创业。牢固树立正确的政绩观多干打基础、利长远、惠百姓的工作绝不干劳民伤财的"面子工程"和"形象工程"说了算、定了干雷厉风行高标准、快节奏地推进工作坚决不当懒官、庸官、太平官。建立政府绩效考核体系把各方面工作引导到科学发展、跨越发展、和谐发展的轨道上来。建立激励导向机制任务完成情况与部门和领导个人评优评先、晋升晋级挂钩真正奖勤奖优严厉惩懒、治庸切实解决干与不干、干好干坏一个样的问题充分调动各级干部干事创业的积极性和主动性。

(三)建设效能政府。转变政府职能促进基本公共服务均等化。深化行政审批制度改革，减少审批事项规范审批行为。加强市县两级行政审批中心建设加大授权力度优化审批流程完善服务方式切实做到"一个窗口对外、一站式办公、一条龙服务"。深入开展"企业服务年"和"项目推进年"活动为企业发展、项目建设提供优质高效服务。完善实施服务承诺制、首问负责制、一次告知两次办结制、超时默许和限时办结制等制度加大行政效能监察和政风行风评议力度建立行为规范、运转协调、办事高效的服务机制。强化目标管理加大督查力度明确职能部门主办责任完善部门间协调配合制推行行政问责制确保政令畅通提高政府执行力。

(四)建设法治政府。严格依照法定权限和程序行使权力、履行职责运用法律手段推进政府工作。健全科学民主决策机制完善社情民意反映制度和重大事项集体决策、专家咨询、社会公示及听证、决策评估等制度。政府及其各部门领导在决策前要深入调研掌握必要的决策知识和依据决不能搞生疏草率、遗患无穷的"拍脑袋"决策。规范行政执法行为落实行政执法责任制和过错追究制做到严格执法、公正执法。深入推行政务公开保证群众知情权、参与权、表达权和监督权。加强行政复议工作。自觉接受人大及其常委会的法律监督、工作监督和人民政协的民主监督办好人大代表议案、建议和政协委员提案。广泛接受社会公众和新闻舆论等方面的监督。

(五)建设廉洁政府。始终牢记"两个务必"坚持勤俭办一切事业珍惜民资民力坚决制止铺张浪费、奢靡之风努力降低行政成本。严格落实廉政建设责任制扎实推进惩治和预防腐败体系建设坚持用制度管权、管事、管人。严格执行领导干部廉洁自律规定做到勤勉尽责、秉公用权。坚持从严治政加强行政监察和审计监督加大案件查办力度对各种违法违纪行为严惩不贷以实际行动取信于民。
